# Exercise Habits in Older Adults: A Scoping Review

**DOI:** 10.3390/healthcare14152375

**Published:** 2026-08-03

**Authors:** Ruotong Peng, Jing Chang, Yishu Zhu, Ronghua Yang, Hui Feng, Yinan Zhao

**Affiliations:** 1 School of Nursing, Chinese Academy of Medical Sciences & Peking Union Medical College, Beijing 100144, China; ruotongpeng@student.pumc.edu.cn; 2 Xiangya School of Nursing, Central South University, Changsha 410013, China; changjing429@163.com (J.C.); 247811038@csu.edu.cn (Y.Z.); feng.hui@csu.edu.cn (H.F.); 3 Department of Nursing, The Fourth Affiliated Hospital of School of Medicine, International School of Medicine, International Institutes of Medicine, Zhejiang University, Yiwu 322000, China; 4 School of Nursing and Institute of Nursing Research, School of Medicine, Zhejiang University, Hangzhou 310058, China; 5 Department of Neonatology, The First Affiliated Hospital of Hunan Traditional Chinese Medical College (Hunan Provincial Directly Affiliated Hospital of Traditional Chinese Medicine), Zhuzhou 412000, China; 13657336427@163.com

**Keywords:** exercise habit, habit formation, physical activity, older adults, scoping review

## Abstract

Exercise habits may support long-term exercise maintenance in older adults, yet the construct has been inconsistently defined and measured. This scoping review examined how exercise habits were conceptualized and operationalized, health-related evidence, and factors reported in relation to exercise initiation and maintenance. PubMed, Embase, Web of Science, EBSCOhost, and the Cochrane Library were searched from inception to July 2025 following PRISMA-ScR guidance, and methodological quality was appraised using the Mixed Methods Appraisal Tool. Of 12,539 records, 25 studies were included. Most studies defined exercise habit through frequency, duration, continuity, participation, or adherence, and none directly assessed cue-dependent automaticity using a validated habit-specific measure. Reported health-related associations were heterogeneous and were based mainly on observational studies and self-reported measures. Recurring factors related to exercise initiation and maintenance were synthesized into a provisional framework comprising initial engagement, routine establishment, and possible habit internalization. By distinguishing behavioral regularity, contextual stability, and automaticity, this review provides a clearer conceptual basis for studying exercise habit formation in older adults. Future longitudinal and intervention studies should assess these components separately and test how cue-based, social, environmental, and program-related supports facilitate exercise habit formation and maintenance in everyday life.

## 1. Introduction

Global population aging has made healthy aging a major public health priority, and exercise is central to preventing functional decline and managing chronic conditions in later life [1,2,3]. However, initiating exercise does not necessarily lead to long-term maintenance [4]. One study reported that only 29.1% of frail older adults exercised regularly [5]. A recent systematic review reported that across 10 studies that defined adherence categorically, the average adherence rate was only 57.5% (range: 21–83%) [6]. Evidence from systematic reviews indicates that completion rates for some exercise programs among older adults can be as low as 65%, with attendance rates at just 58% [7]. The frequency of self-directed home-based exercise was only 1.5 to 3 sessions per week, well below recommended health guidelines [8]. Identifying approaches that help older adults continue exercising after professional supervision or structured programs have ended is therefore an important clinical and public health challenge.

Given that reliance on conscious motivation and deliberate effort often wanes over time, a key to overcoming this problem of long-term maintenance may lie in the power of habits. Based on the classical habit theory proposed by Wood and Rünger [9], habit formation involves a complex interactive process with the goal system. Habits initially emerge from explicit goals (e.g., pursuing health) and are formed through repetition in stable contexts. Once established, these behaviors can be automatically activated by contextual cues (e.g., time and location) and regulated and modified by current goals. Furthermore, the sense of accomplishment and positive self-attribution that individuals derive from sustained exercise reinforce their initial goals, forming a consolidated habit-goal cycle.

Previous studies suggest that stable routines and habit-related processes may contribute to exercise maintenance among older adults [10,11,12]. Nevertheless, existing reviews have mainly examined adherence to prescribed exercise programs [6,7], the health benefits of exercise [13], or habit formation across broader behaviors and populations [12]. Specifically, Singh et al. [12] examined the time required to form a habit and its determinants across multiple health behaviors and populations, rather than how exercise habit has been conceptualized, measured, and maintained specifically among older adults. Consequently, it remains unclear whether the available literature on older adults primarily reflects regular exercise participation, long-term behavioral maintenance, program adherence, psychological automaticity, or a combination of these constructs. Evidence concerning exercise initiation and maintenance is also distributed across heterogeneous quantitative and qualitative studies. To address these gaps, the present review focuses specifically on older adults, distinguishes regular or sustained exercise behavior from cue-dependent habit automaticity, and integrates quantitative and qualitative evidence concerning exercise initiation and long-term maintenance. This distinction has practical relevance because strategies that support attendance or adherence during supervised exercise programs may differ from those needed to integrate exercise into stable daily routines as professional support is gradually reduced or withdrawn.

Scoping reviews are particularly appropriate when the purpose is to map the breadth and characteristics of an evidence base, clarify key concepts and definitions, examine how research has been conducted, and identify knowledge gaps [14,15]. This approach was well suited to the present topic because the literature on exercise habits among older adults uses inconsistent terminology and operational definitions and encompasses diverse study designs, populations, measurement approaches, and health-related outcomes. Rather than evaluating the effectiveness of a specific intervention or estimating a pooled effect, this review mapped the scope, conceptualization, measurement, and characteristics of the available evidence. Accordingly, the review was guided by the following research questions: (1) How have exercise habits among older adults been conceptualized, defined, and measured? (2) What health-related outcomes have been associated with exercise habits among older adults? (3) What factors and processes have been reported in relation to the formation and maintenance of exercise habits in this population?

## 2. Materials and Methods

### 2.1. Search Strategy

The review was registered in advance at the Open Science Framework website (DOI 10.17605/OSF.IO/2M6VN). A literature search was conducted in PubMed, Web of Science, Embase, EBSCOhost, and the Cochrane Library databases to identify relevant English-language articles (end-of-search date: July 2025). Further relevant publications were identified through the reference lists of the selected publications. All search algorithms were modified and refined for each database. The PubMed search strategy was as follows: ((“older adult*”[Title/Abstract] OR “older people”[Title/Abstract] OR “elderly”[Title/Abstract] OR “older population”[Title/Abstract] OR “senior citizen*”[Title/Abstract]) AND (“Exercise”[Mesh] OR “exercise”[Title/Abstract] OR “exercises”[Title/Abstract] OR “aerobic exercise”[Title/Abstract] OR “resistance exercise”[Title/Abstract] OR “multi-component exercise”[Title/Abstract] OR “Tai Chi”[Title/Abstract] OR “swim”[Title/Abstract] OR “Yoga”[Title/Abstract])) AND (“Habits”[Mesh] OR “Habits”[Title/Abstract] OR “Habit”[Title/Abstract] OR “long-term”[Title/Abstract] OR “routine”[Title/Abstract]) AND ((humans[Filter]) AND (English[Filter])). The search syntax was adapted to suit each database. The benchmark definitions and search strings are provided in Supplementary Table S1.

### 2.2. Inclusion and Exclusion Criteria

Inclusion criteria: (1) Studies with mixed-age populations were required to provide stratified data for the subgroup aged ≥60 years. (2) Concept: Studies were eligible if they explicitly examined exercise habits, habitual exercise, or regular exercise as a repeated or sustained behavior. Because terminology and measurement were inconsistent across the literature, eligible operationalizations were classified into two categories: regularity-based operationalizations, in which exercise habit was defined using indicators such as exercise frequency, duration, continuity, or long-term participation, and automaticity-based operationalizations, in which habit strength or automaticity was assessed using a validated instrument. Author-defined measures of “regular” or “habitual” exercise were included in the first category only when they reflected repeated or sustained behavior rather than occasional participation. These operationalizations were included to map the range of definitions used in the literature and were not assumed to be equivalent or interchangeable measures. (3) Context: Peer-reviewed journal publications (cross-sectional, longitudinal, qualitative, and quantitative) in English without date limiters until the final search date. Cross-sectional studies were required to examine associations between exercise habits and health/behavioral outcomes. In contrast, longitudinal/intervention studies were required to demonstrate either habit formation processes or ≥3 months of post-intervention exercise maintenance.

Exclusion criteria: (1) Studies that conflate exercise habits with occasional activity (e.g., one-time interventions or surveys without assessing behavioral consistency). (2) Studies that reported only post-intervention physical or cognitive outcomes without assessing exercise regularity, maintenance, adherence, or habit-related processes.

### 2.3. Study Selection

The screening process was conducted using EndNote 21 software (Clarivate, Philadelphia, PA, USA). Initially, two authors (R.P. and J.C.) screened all study titles and abstracts for eligibility according to the study criteria. Next, the two authors (R.P. and J.C.) checked all full-text articles to determine whether they included the exercise habit concept and were related to older adults (≥60). In the event of any disagreement, a third researcher (Y.Z.) was invited to resolve it.

### 2.4. Data Extraction

Study author, year, country, target population, aim, design, exercise type, operational definition and measurement of exercise habit, operationalization category, and reported outcomes were extracted and coded using Microsoft Excel for Microsoft 365 (Microsoft Corporation, Redmond, WA, USA). For quantitative studies, we additionally extracted comparison groups, the most fully adjusted effect estimates, 95% confidence intervals, p values, covariate adjustment, and whether findings were favorable, null, adverse, or mixed. Two authors (R.P. and J.C.) independently selected and extracted the data. Disagreements were resolved through consultation with a third author (Y.Z).

### 2.5. Critical Appraisal of Included Studies

The methodological quality of all included studies was appraised using the 2018 Mixed Methods Appraisal Tool (MMAT) [16]. The MMAT was selected because it provides design-specific criteria for qualitative, quantitative, and mixed-methods studies. Two reviewers (R.P. and J.C.) independently assessed each study using the criteria corresponding to its design, with each item rated as “Yes,” “No,” or “Can’t tell”. Disagreements were resolved through discussion with a third reviewer (Y.Z.). The appraisal was not used as an exclusion criterion. Instead, studies with concerns regarding representativeness, exposure measurement, non-response or attrition, and confounding control were interpreted more cautiously and were not used to support strong temporal or causal conclusions. Cross-sectional studies were treated as evidence of reported associations or candidate factors rather than as evidence of temporal relationships. In contrast, longitudinal findings were considered more informative regarding temporality, although residual confounding remained possible. In accordance with MMAT guidance, no overall numerical score was calculated, and studies were not weighted numerically based on their appraisal results.

### 2.6. Data Synthesis

Owing to heterogeneity in the operational definitions of exercise habit, study designs, populations, and outcomes, the findings were synthesized descriptively and interpretively, and no meta-analysis was performed. First, definitions and measurement approaches were mapped according to whether they assessed behavioral regularity or habit strength/automaticity. Second, quantitative findings were grouped by study design and health-outcome domain, including physical, psychological, cognitive, and social outcomes. Cross-sectional, longitudinal, and intervention-related findings were summarized separately. Within each design stratum, findings were further compared according to the exercise-habit operationalization categories assigned in this review, as detailed in Supplementary Tables S2B and S4A,B. Effect estimates were not pooled or directly compared across heterogeneous studies. Qualitative and mixed-methods findings on exercise initiation and maintenance were compared across studies and grouped into recurring motivational, contextual, behavioral, social, and reinforcing factors. These findings informed a proposed conceptual map comprising initial engagement, routine establishment, and possible habit internalization. The critical appraisal informed the interpretation of the evidence, although studies were not numerically weighted according to their appraisal results.

## 3. Results

### 3.1. Study Selection and Characteristics

**Figure 1:**
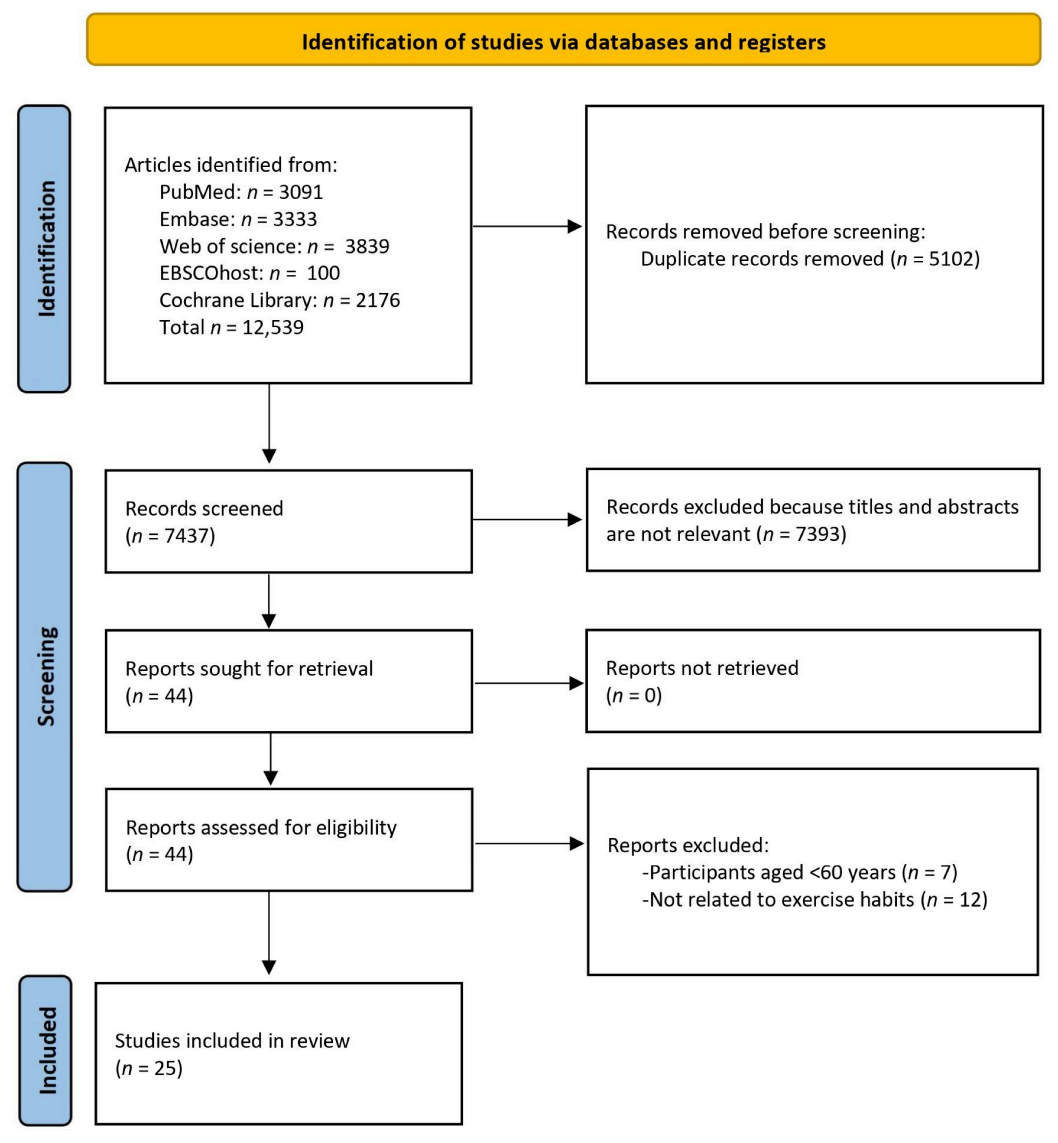
PRISMA flow diagram of study selection.

**Figure 2:**
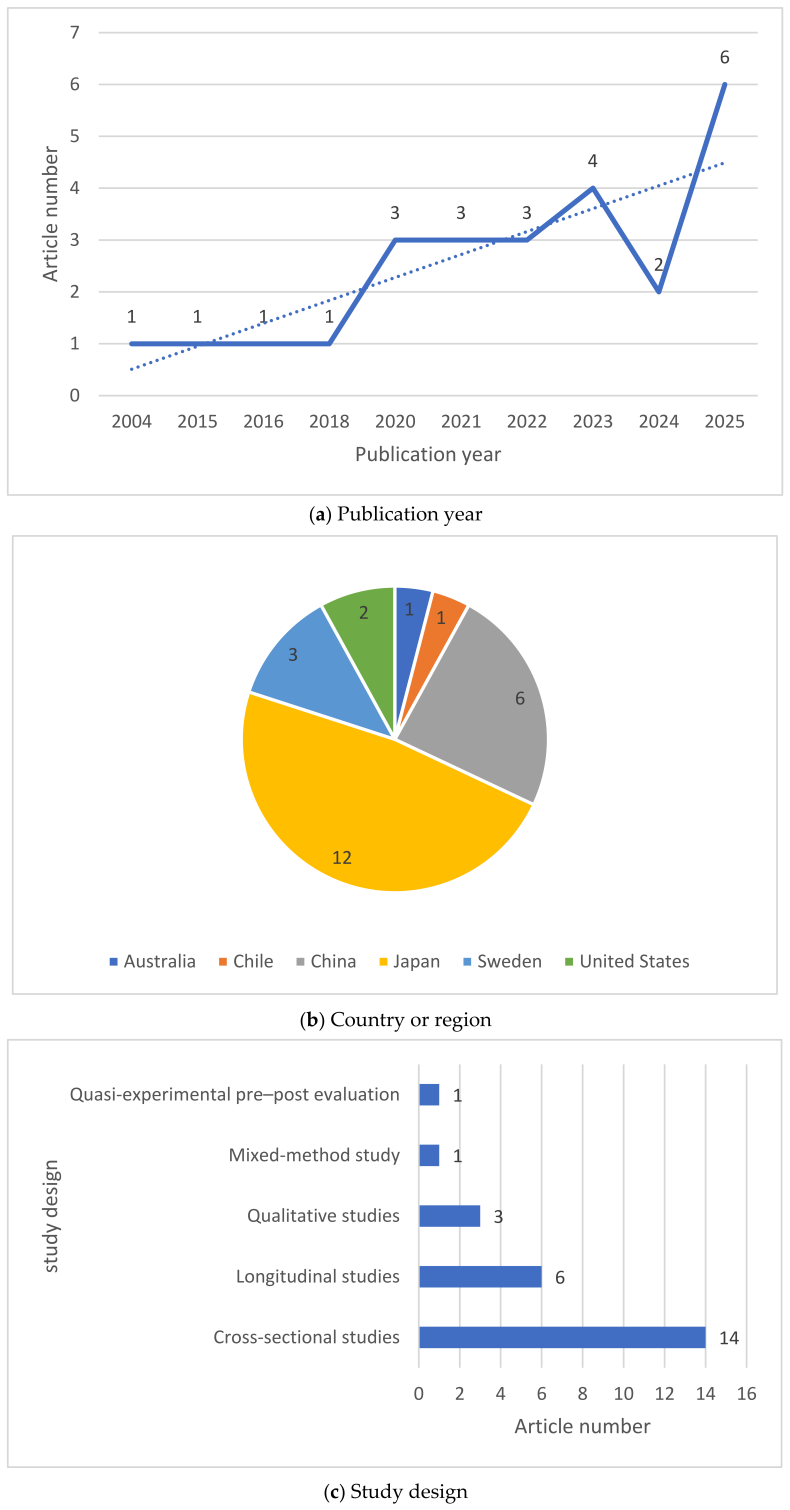
Characteristics of the included studies. Panel (**a**) shows publication year; the solid line represents the annual number of publications, and the dotted line represents the linear trend. Panel (**b**) shows country or region, and panel (**c**) shows study design among the 25 included studies.

A total of 12,539 articles were initially retrieved. After duplicates were removed, 7437 records remained, of which 7393 were excluded after title and abstract screening. Finally, 44 articles were assessed in full, of which 25 were included in the review [17,18,19,20,21,22,23,24,25,26,27,28,29,30,31,32,33,34,35,36,37,38,39,40,41]. The study-selection process is shown in Figure 1. The studies were published from 2004 to 2025, and the number of studies by year of publication is shown, with the majority being published in 2025 (*n* = 6), followed by 2023 (*n* = 4). The studies were conducted in six countries, predominantly in Japan (*n* = 12), followed by China (*n* = 6), Sweden (*n* = 3), the United States (*n* = 2), Australia (*n* = 1), and Chile (*n* = 1). The study designs included 14 cross-sectional studies; six longitudinal studies comprising five cohort or follow-up studies and one longitudinal intervention follow-up; three qualitative studies; one mixed-methods study; and one quasi-experimental pre–post program evaluation. Figure 2 summarizes the publication years, geographical distribution, and study designs. A concise summary of the study populations, exercise-habit operationalizations, outcome domains, and main findings is provided in Table 1. Detailed study characteristics and measurement approaches are presented in Supplementary Table S2A,B.

### 3.2. Methodological Quality of the Included Studies

Detailed results of the MMAT appraisal are presented in Supplementary Table S3A–D. Across the quantitative studies, recurring methodological concerns included limited participant representativeness, reliance on self-reported and non-standardized measures of exercise habits, incomplete reporting of non-response or attrition, and residual confounding. The predominance of cross-sectional designs also limited conclusions regarding temporal direction. The qualitative studies provided relevant contextual evidence, although the coherence of sampling, analysis, and interpretation was not always fully reported. The mixed-methods study showed limited explicit integration between its quantitative and qualitative components. Accordingly, findings from studies with these methodological limitations were treated as exploratory or associative evidence and were not used to establish temporal or causal relationships. The appraisal findings also supported presenting the conceptual map as a provisional interpretation rather than a validated process model.

### 3.3. Conceptualization of Exercise Habits

The included studies used heterogeneous approaches to conceptualize and operationalize exercise habits. Twenty-two of the 25 included studies assessed exercise regularity, participation, or maintenance using indicators such as weekly frequency, session duration, continuity, adherence, activity level, or self-reported regular exercise. The remaining three qualitative studies explored processes related to exercise initiation and maintenance without using a formal habit measure. None of the included studies assessed habit strength or automaticity using a validated habit-specific instrument. Operational criteria varied considerably. For example, Xu et al. [40] defined exercise habit as exercising for at least 30 min per session on at least three days per week, whereas Nakajima et al. [32] assessed participation across 14 exercise types during the previous year without specifying minimum frequency or duration. Hayashi et al. [24] assessed weekly home-exercise frequency using a single item. Some studies additionally required sustained participation over several months or at least one year, while others assessed adherence to a structured exercise program. Therefore, the available evidence predominantly reflected regular or sustained exercise behavior rather than directly measured psychological habit automaticity. The specific definitions, measurement tools or survey items, and operationalization categories used in each study are presented in Supplementary Table S2B.

### 3.4. Association Between Exercise Habits and Health Outcomes

Most included studies operationalized exercise habit using indicators of regularity, continuity, or sustained participation. Accordingly, the health-related findings summarized below primarily reflect these behavioral operationalizations. No included study directly assessed the automaticity component of exercise habit using a validated habit-specific measure. Longitudinal studies generally reported associations with lower mortality and frailty, although the findings were not uniform. In a national Chinese cohort, sustained exercise and transition from inactivity to activity were associated with lower all-cause mortality compared with sustained inactivity (adjusted HR 0.74, 95% CI 0.66–0.83; and HR 0.88, 95% CI 0.80–0.97, respectively), whereas transition from activity to inactivity was not significantly associated with mortality [27]. Pre-admission exercise was associated with lower post-discharge mortality among older patients with heart failure (adjusted HR 0.75, 95% CI 0.58–0.98) [31]. Regular exercise, as defined in the original study, was associated with a lower incidence of frailty over 7 years. Still, this association did not differ significantly from that of moderate-to-vigorous non-exercise physical activity [29]. Similarly, exercising with others was associated with lower risks of disability and mortality than not exercising, whereas adjusted comparisons with exercising alone were not statistically significant [21].

Cross-sectional studies reported associations across physical, psychological, cognitive, and social outcomes, but several findings were sex- or outcome-specific. Longer exercise duration was associated with lower frailty prevalence [28], and physically inactive older women had less favorable cardiometabolic, body composition, and physical performance profiles [25]. Exercise participation was associated with lower loneliness among men (OR 0.75, 95% CI 0.65–0.88) but not women (OR 0.96, 95% CI 0.82–1.13) [32]. Life-course exercise patterns were associated with some favorable musculoskeletal, cognitive, and depressive-symptom outcomes, although associations varied by sex and specific outcome; several osteoporosis, brain-volume, and biomarker comparisons were not statistically significant [33,35,36,38]. One quasi-experimental study reported improvements in mobility and cognition after 6 months, but the absence of a control group limited the attribution of these changes to the intervention [23].

Overall, the available evidence suggested associations between regular or sustained exercise and multiple health-related outcomes. Still, the magnitude and consistency of these associations varied across populations, definitions, and study designs. Longitudinal studies provided stronger evidence of temporal associations than cross-sectional studies, but residual confounding and reverse causation could not be ruled out. Comparisons across operationalization categories were not possible because no included study directly assessed automaticity using a validated habit-specific instrument. Findings among studies using behavioral operationalizations also remained heterogeneous across populations and outcomes. Detailed effect estimates; confidence intervals; operationalization categories; and favorable, null, adverse, and mixed findings are presented in Supplementary Table S4A,B.

### 3.5. Proposed Interpretive Conceptual Map of Exercise Habit Formation in Older Adults

**Figure 3:**
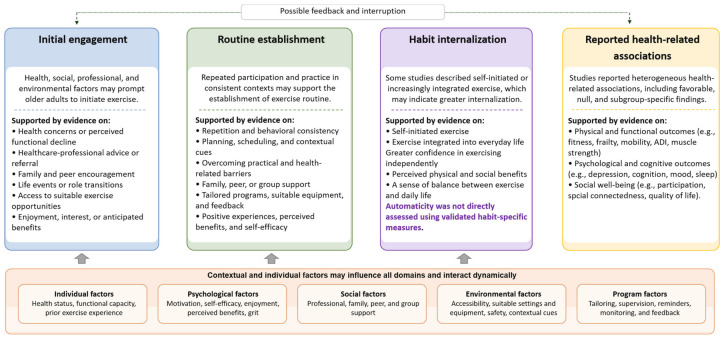
Proposed interpretive conceptual map of exercise initiation, routine establishment, and habit internalization in older adults. The map organizes recurring findings across the included studies into three overlapping domains. Arrows indicate hypothesized relationships and feedback only and do not represent an established temporal sequence or causal pathway.

An interpretive synthesis of the included studies identified recurring findings concerning initial engagement, routine establishment, and possible habit internalization (Figure 3). This conceptual map represents a proposed interpretation of the available evidence rather than an empirically established sequence or causal model. No included study evaluated these domains as a complete pathway, and their relationships may overlap, interact, or be interrupted.

### 3.6. Factors Related to Initial Engagement

The included studies reported several factors in relation to exercise initiation. Perceived threats to autonomy, such as fear of falling or functional decline, were reported as reasons for initiating exercise [34], aligning with core motivators such as health preservation and maintenance of independence [22]. Furthermore, the expectation of future rewards, such as setting a positive example for one’s children, weight management, and social engagement, has been identified as a reported motivator for initiating exercise, with the understanding that these benefits will reinforce long-term participation [22]. Externally, referrals and encouragement from healthcare professionals were reported as important prompts for participation [41]. Among older adults with type 1 diabetes, glucose-monitoring technology was reported to support risk management when deciding whether and how to exercise [19].

### 3.7. Factors Related to Routine Establishment

Continued exercise was reported alongside several behavioral and contextual strategies. Participants described strategies such as stimulus control, which involved modifying the environment, and counterconditioning, which involved replacing sedentary behaviors with active alternatives [22]. Social and group-based exercise settings were frequently reported as associated with continued participation, although adjusted comparisons between exercising with others and alone were not consistently significant [21,22,30]. Tailored programs, suitable equipment, supportive exercise settings, and regular assessment were reported to support continued participation [41]. One follow-up study also reported that physical activity was maintained 7 months after a structured multicomponent program [18]. Digital reminders and self-monitoring were reported as strategies for supporting self-managed exercise [34]. Greaney et al. [22] also demonstrated exercise commitment and used experiential processes to maintain exercise. For example, self-reevaluation emerged when participants stated that they felt “guilty” when they did not exercise.

### 3.8. Factors Potentially Related to Habit Internalization

Some studies described experiences that may reflect a greater integration of exercise into everyday life, including self-initiated participation, perceived physical and social benefits, greater confidence in exercising independently, and a sense of balance between exercise and other daily priorities [34,41]. Determination, grit, and exposure to exercise-supportive social networks were also reported to be associated with continued exercise or longer maintenance of self-directed exercise [22,26,37]. However, none of these studies directly assessed cue-dependent automaticity using a validated habit-specific instrument. These findings may therefore indicate greater internalization, but they do not confirm that an automatic exercise habit had developed.

## 4. Discussion

This scoping review identified three principal findings. First, the term “exercise habit” was used inconsistently across the 25 included studies. Most studies operationalized it through exercise frequency, duration, continuity, participation, or adherence, whereas none directly assessed cue-dependent automaticity using a validated habit-specific instrument. Second, several studies reported associations between regular or sustained exercise and physical, psychological, cognitive, and social outcomes, although the findings were heterogeneous, including null, subgroup-specific, and attenuated associations. Third, recurring findings concerning exercise initiation, routine establishment, and possible internalization were organized into a proposed conceptual map. The MMAT appraisal nevertheless identified recurring concerns regarding self-reported and non-standardized exposure measures, participant representativeness, incomplete reporting of non-response or attrition, and residual confounding. These limitations, together with the predominance of cross-sectional evidence, require cautious interpretation. The findings represent reported associations and experiences rather than confirmed effects or causal relationships.

The conceptual heterogeneity identified in this review has important implications for interpreting the evidence. Habit theory suggests that repeated behavior may facilitate the development of cue-dependent automaticity, but frequency and continuity alone do not establish that a behavior has become habitual in the psychological sense [9,42,43]. The included studies therefore captured different aspects of exercise habit, ranging from repeated participation and long-term maintenance to adherence within structured programs. This variation may partly explain differences in reported associations and limit direct comparisons across studies. Future research should measure exercise participation and habit strength separately, using behavioral indicators alongside validated habit-specific instruments, rather than treating regularity and automaticity as interchangeable constructs [42,44].

The way physical activity and exercise were assessed also affects the interpretation of the evidence. Most included studies relied on self-reported questionnaires, single items, or retrospective reports of exercise frequency, duration, participation, or continuity. In contrast, only a small number used program adherence or participation records. Although self-reported measures are practical, physical activity questionnaires for older adults show variable measurement properties and may be affected by recall and reporting errors [45]. Adherence or participation records provide more direct information about exercise completed within structured programs, but they may not capture unsupervised exercise in daily life. Device-based measures such as accelerometers can provide more objective information on the amount, frequency, duration, and temporal patterns of physical activity. However, their validity depends on device placement, calibration, and data-processing methods [46]. More importantly, neither self-reported nor device-based behavioral measures alone can establish cue-dependent habit automaticity [42,44]. Future studies should therefore combine behavioral measures with information on contextual stability and validated habit-specific assessments of automaticity.

The heterogeneity of health-related findings may partly reflect differences in the individual and social contexts in which exercise is initiated and maintained. Several longitudinal studies reported favorable associations between regular or sustained exercise and mortality, frailty, or functional outcomes, although residual confounding, exposure misclassification, and baseline health differences remain possible [29,31]. The absence of a clear advantage for exercising with others over exercising alone suggests that social context may not operate uniformly [21]. Findings also differed between men and women across outcome domains. Exercise participation was associated with lower loneliness in men but not in women, whereas life-course exercise showed clearer associations with bone outcomes in women [32,33]. Previous research has shown that older men and women may differ in their motivations for physical activity and preferred exercise contexts, including social interaction, supervision, and the characteristics of exercise partners [47,48]. Such differences may shape initial engagement and continued participation and may partly account for variation across social and physical outcomes. However, the included studies did not directly examine the mechanisms underlying these differences, and most were cross-sectional. Future prospective studies should examine whether motivation, social support, exercise type, and exercise context contribute to differences between men and women in exercise initiation, maintenance, and related outcomes.

The conceptual map integrates factors directly reported across the included studies, including health concerns, professional encouragement, social support, repeated participation, perceived benefits, and the integration of exercise into daily life [22,34,41]. Their organization into initial engagement, routine establishment, and possible habit internalization represents an interpretive synthesis rather than a pathway evaluated in a single study. Cross-sectional designs cannot establish temporal relationships among these domains. In addition, non-standardized measures may classify regular participation or adherence as habit formation without demonstrating automaticity. The map consequently provides a provisional organizing framework for hypothesis generation and future research rather than a validated developmental sequence or causal model.

Distinguishing regular exercise participation from habit automaticity also has practical implications for long-term exercise maintenance. Strategies that support attendance or adherence during supervised programs may not be sufficient to help older adults integrate exercise into stable daily routines. The factors identified in the conceptual map may therefore be relevant to the transition from supervised rehabilitation to sustained home-based exercise. Clinicians and exercise professionals could help patients select one safe, feasible, and personally meaningful exercise and link it to a stable daily cue. For example, balance exercises could be performed after breakfast, strengthening exercises after taking morning medication, or walking after a regularly scheduled activity. Repeating the same behavior in a consistent context may help establish a stable routine, particularly when the plan is simple and easy to perform [43]. Professional guidance and individualized programs may provide structure during the early stages of maintenance. Suitable equipment, visible reminders, and family or peer support may offer additional assistance [41]. However, regular adherence should not be assumed to indicate automaticity. External support may be gradually reduced while clinicians continue to assess safety, contextual stability, functional capacity, and the conscious effort required to initiate exercise [44].

Digital tools may support home-based exercise through reminders, exercise demonstrations, progress tracking, and remote feedback. However, their value is likely to depend on whether they can be integrated into stable daily routines rather than used as isolated prompts [34,49]. Digital approaches may also be less accessible to older adults with limited digital literacy, sensory or cognitive impairments, restricted device access, or inadequate caregiver support [50]. Low-technology alternatives, including printed exercise plans, calendars, visible equipment, and telephone follow-up, should therefore remain available. Future longitudinal and intervention studies should assess exercise behavior, contextual stability, and habit automaticity separately and repeatedly over time. Such studies are needed to determine whether cue-based strategies can support exercise maintenance and whether the proposed conceptual domains are practically relevant across diverse clinical and cultural settings.

## 5. Limitations

Several limitations should be considered. Most included studies were cross-sectional, which prevented conclusions about temporal order and raised the possibility of reverse causation. Exercise habit was defined using heterogeneous and predominantly self-reported measures, and no study used a validated habit-specific instrument to assess automaticity. Differences in exposure definitions, populations, and outcomes limited direct comparison across studies. The evidence was also geographically concentrated, with 12 of the 25 studies conducted in Japan, which may restrict transferability to other cultural and healthcare settings. Restricting the search to English-language peer-reviewed publications may have excluded relevant regional and grey literature. Although methodological quality was appraised using the MMAT, the appraisal identified recurring concerns related to representativeness, measurement validity, missing data, and residual confounding. Statistical pooling and formal assessment of publication bias were not feasible because of the heterogeneity of the evidence.

## 6. Conclusions

This scoping review shows that research on exercise habits in older adults has largely assessed regular or sustained participation rather than cue-dependent automaticity, and that reported health-related associations remain heterogeneous. By distinguishing behavioral regularity, contextual stability, and automaticity, this review offers a provisional framework comprising initial engagement, routine establishment, and possible habit internalization. Future longitudinal and intervention studies should test these components separately and determine how cue-based, social, environmental, and program-related supports can facilitate exercise habit formation and maintenance in everyday life.

## Figures and Tables

**Table 1:** Summary of the included studies.

Study	Design and Sample	Operationalization Category and Definition/Measure	Outcome(s) and Main Finding
Arkkukangas et al., 2018 [17]	Longitudinal intervention follow-up; *n* = 114; community-dwelling adults aged ≥75 years.	Regularity-/maintenance-based: Baseline activity habits as a predictor; 52-week exercise and walking adherence records.	Adherence—Baseline activity habits predicted exercise and walking adherence; motivational interviewing was also associated with higher exercise adherence.
Arkkukangas et al., 2022 [18]	Prospective follow-up; *n* = 200; community-dwelling older adults (mean age ≈ 72 years).	Regularity-/maintenance-based: Six-level Frändin–Grimby Activity Scale, from hardly any activity to strenuous regular exercise.	Activity maintenance—Baseline activity level, self-rated health, and higher-challenge group participation were associated with activity level at 7 months.
Chakrabarti et al., 2022 [19]	Cross-sectional survey; *n* = 30; adults aged ≥60 years with type 1 diabetes using insulin pumps.	Regularity-/maintenance-based: Exercise days/week, session duration, and activity patterns; no binary habit definition.	Participation and self-management—Median 4 exercise days/week and 60 min/session; glucose concerns influenced participation and management practices.
Chen et al., 2025 [20]	Mixed-methods study; *n* = 280 survey participants and 5 interviewees; community-dwelling adults aged ≥60 years.	Regularity-/maintenance-based: Exercise habit (yes/no), frequency (<3 vs. ≥3 times/week), duration (<30 vs. ≥30 min/session), and type were assessed separately.	Participation and preferences—Chronic pain was associated with lower participation at ≥3 times/week, whereas overall habit status and session duration were not significant; walking and social/environmental support were commonly reported.
Fujii et al., 2020 [21]	Prospective 5-year cohort; *n* = 1520; community-dwelling adults aged ≥65 years.	Regularity-/maintenance-based: Exercise ≥ 1 time/week; exercisers classified as exercising alone or with others.	Disability and mortality—Exercising with others versus no exercise was associated with lower risks; adjusted comparisons with exercising alone were not significant.
Greaney et al., 2004 [22]	Qualitative focus groups; *n* = 29; adults aged ≥60 years.	Qualitative process evidence (no formal habit measure): No formal frequency, duration, or automaticity threshold; qualitative process evidence.	Initiation and maintenance—Health maintenance, independence, and fear of illness were reported as motivators; strategies included counterconditioning, helping relationships, stimulus control, and self-liberation.
Hau et al., 2016 [23]	Quasi-experimental pre–post evaluation; *n* = 50; Chinese older adults in the United States (mean age 68.4 years).	Regularity-/maintenance-based: Program goal ≥ 3 sessions/week; adherence assessed during the 6-month program.	Adherence and health outcomes—Seventy-eight percent met the exercise goal; mobility, cognition, executive function, and depressive symptoms improved from baseline, but there was no comparison group.
Hayashi et al., 2022 [24]	Cross-sectional survey; *n* = 1103; community-dwelling adults aged ≥65 years.	Regularity-/maintenance-based: Weekly home-exercise frequency was measured using one self-reported item.	Social frailty and depression—Social frailty was associated with depressive symptoms among those not exercising at home, but not among home exercisers.
Hernandez-Martinez et al., 2023 [25]	Analytical cross-sectional study; *n* = 179; women aged ≥60 years.	Regularity-/maintenance-based: Physically active versus inactive according to weekly moderate- or vigorous-intensity activity recommendations.	Physical and cardiometabolic outcomes—Inactive participants had less favorable cardiometabolic, body-composition, grip-strength, mobility, and gait profiles.
Kawasaki et al., 2020 [26]	Cross-sectional study; *n* = 59; community-dwelling adults aged ≥65 years with low back pain.	Regularity-/maintenance-based: Moderate-or-higher intensity exercise > 2 times/week and >30 min/session; maintenance duration recorded.	Exercise maintenance—Grit was positively correlated with the duration of self-directed exercise and remained associated with duration in multivariable analysis.
Li et al., 2025 [27]	National longitudinal cohort; *n* = 7231; adults aged ≥60 years.	Regularity-/maintenance-based: Repeated self-report classified as inactive, active, inactive-to-active, or active-to-inactive.	Mortality—Sustained activity and transition from inactivity to activity were associated with lower mortality; findings varied by sleep duration and sex.
Lin et al., 2025 [28]	Cross-sectional study; *n* = 2545; adults aged ≥65 years.	Regularity-/maintenance-based: Exercise regularity, type, days/week, and daily duration.	Frailty—Longer daily exercise duration was associated with lower frailty; absence of aerobic exercise was associated with higher pre-frailty.
Lyu et al., 2025 [29]	Prospective 7-year cohort; *n* = 1288; community-dwelling adults aged ≥65 years.	Regularity-/maintenance-based: Exercise ≥ 30 min/day, >2 times/week, and continued for >1 year.	Frailty—Exercise habit and moderate-to-vigorous non-exercise activity were each associated with lower prevalent and incident frailty; their estimates did not differ significantly.
Makino et al., 2015 [30]	Cross-sectional questionnaire study; *n* = 304; community-dwelling older adults (mean age 70.3 years).	Regularity-/maintenance-based: Exercise > 3 times/week for ≥30 min/session; individual-versus group-based setting.	Psychological and dietary outcomes—Group-based exercisers had more favorable depressive-symptom and self-efficacy measures; the adjusted dietary-variety association was not significant.
Nakade et al., 2025 [31]	Longitudinal cohort/post hoc analysis; *n* = 1262; hospitalized adults aged ≥65 years with heart failure.	Regularity-/maintenance-based: Usual-week walking/moderate activity ≥ 30 min and vigorous activity ≥ 20 min before admission.	Function and mortality—Pre-admission exercise was associated with better physical function and lower adjusted post-discharge mortality.
Nakajima et al., 2025 [32]	Cross-sectional study; *n* = 10,152; community-dwelling adults aged ≥65 years.	Regularity-/maintenance-based: Participation in 14 exercise types during the previous year; no minimum frequency or duration.	Loneliness—Exercise participation was associated with lower loneliness among men but not among women; the association differed by exercise type.
Otsuka et al., 2021 [33]	Cross-sectional study; *n* = 1596; adults aged 65–84 years.	Regularity-/maintenance-based: Adolescent sports-club participation and current exercise habit (yes/no).	Bone outcomes—Women active in both periods had higher bone mineral density and lower osteoporosis prevalence; findings in men were limited.
Pettersson et al., 2023 [34]	Qualitative grounded-theory study; *n* = 18; adults aged ≥70 years who maintained digital fall-prevention exercise.	Qualitative process evidence (no formal habit measure): ≥60 min/week during the final 3 intervention months; habit development explored qualitatively.	Self-managed maintenance—Participants described acting against threats, coordinating routines, using cues and evaluation, and maintaining balance between exercise and daily life.
Shi et al., 2024 [35]	Cross-sectional study; *n* = 1615; adults aged 65–84 years.	Regularity-/maintenance-based: Adolescent sports-club participation and current exercise habit (yes/no).	Cognition and biomarkers—Activity in both periods was associated with lower odds of MCI; brain-volume and biochemical-marker findings were not significant.
Shi et al., 2024 [36]	Cross-sectional study; *n* = 1629; adults aged 65–84 years.	Regularity-/maintenance-based: Adolescent sports-club participation; current exercise > 2 times/week for ≥30 min/session.	Depressive symptoms—Exercise in adolescence, old age, or both was associated with lower prevalence in men and women, without a clear additive pattern.
Sugisawa et al., 2020 [37]	Cross-sectional survey with retrospective life-course recall; *n* = 739; adults aged ≥65 years.	Regularity-/maintenance-based: Exercise > 30 min twice/week or >20 min three times/week for >1 year.	Exercise maintenance—Life-course exercise habits in social networks were associated with participants’ own late-life exercise habits.
Tabata et al., 2023 [38]	Cross-sectional study; *n* = 1607; adults aged 65–84 years.	Regularity-/maintenance-based: Adolescent sports-club participation; current exercise > 2 times/week for ≥30 min/session.	Sarcopenia—Men active in both periods had lower risks of sarcopenia, low muscle mass, and low performance; women had a lower risk of low muscle performance only.
Wang S. et al., 2021 [39]	Cross-sectional path analysis; *n* = 338; nursing-home residents aged ≥60 years.	Regularity-/maintenance-based: Regular exercise ≥ 30 min/day on ≥3 days/week.	MCI pathways—Direct and indirect associations through frailty, depression, sleep quality, and social support were reported; causal direction could not be established.
Xu et al., 2023 [40]	Cross-sectional study; *n* = 370; adults aged ≥60 years.	Regularity-/maintenance-based: Exercise ≥ 30 min/session on ≥3 days/week.	Sleep—Regular exercise was associated with a higher likelihood of good sleep; age, pain, and self-rated health were also associated with sleep quality.
Wang Y.F. et al., 2021 [41]	Qualitative phenomenological study; 21 older exercisers and 2 instructors.	Qualitative process evidence (no formal habit measure): No formal frequency, duration, or automaticity threshold; participants had continued-exercise experience.	Initiation and maintenance—Doctor referral, tailored programs, suitable equipment, a supportive climate, and regular assessment were reported alongside self-initiated exercise, perceived functional benefits, and wider social networks.

Note: Twenty-two studies assessed exercise regularity, participation, maintenance, or adherence. Three qualitative studies examined processes related to exercise initiation and maintenance without using a formal habit measure. No included study directly assessed cue-dependent habit automaticity using a validated habit-specific instrument.

## Data Availability

All data extracted and analyzed in this scoping review are available within the article and Supplementary Materials.

## Supplementary Materials

The following supporting information can be downloaded at https://www.mdpi.com/article/10.3390/healthcare14152375/s1, Table S1: Search strategies across PubMed, Embase, Web of Science, EBSCOhost, and the Cochrane Library; Table S2A: Characteristics of the included studies; Table S2B: Definitions and measurement approaches used to operationalize exercise habit; Table S3A–D: Methodological quality appraisal using the Mixed Methods Appraisal Tool; Table S4A: Longitudinal, follow-up, and quasi-experimental quantitative findings; Table S4B: Cross-sectional and mixed-methods quantitative findings; Checklist S1: PRISMA-ScR checklist.

## Abbreviations

The following abbreviations are used in this manuscript:

COVID-19	Coronavirus disease 2019
HR	Hazard ratio
MCI	Mild cognitive impairment
MMSE	Mini-Mental State Examination
MVPA	Moderate-to-vigorous physical activity
NEPA	Non-exercise physical activity
OSF	Open Science Framework
PRISMA-ScR	Preferred Reporting Items for Systematic Reviews and Meta-Analyses Extension for Scoping Reviews
SPPB	Short Physical Performance Battery
SRHI	Self-Report Habit Index
