# Supplementary material for: Exercise Habits in Older Adults: A Scoping Review
Source: Healthcare (Basel). 2026 Aug 3;14(15):2375. doi: 10.3390/healthcare14152375 (PMC13463881; doi:10.3390/healthcare14152375)
Supplement: Supplementary file 1 [file healthcare-14-02375-s001.zip › healthcare-4435343-supplementary.pdf]

Supplementary Table S1. Search strategies across databases (total records: 12,539)

Database 1: PubMed

Run date: 15/07/2025

(("older adult*"[Title/Abstract] OR "older people"[Title/Abstract] OR "elderly"[Title/Abstract] OR "older population"[Title/Abstract] OR "senior citizen*"[Title/Abstract]) AND ("Exercise"[Mesh] OR "exercise"[Title/Abstract] OR "exercises"[Title/Abstract] OR "aerobic exercise"[Title/Abstract] OR "resistance exercise"[Title/Abstract] OR "multi-component exercise"[Title/Abstract] OR "Tai Chi"[Title/Abstract] OR "swim"[Title/Abstract] OR "Yoga"[Title/Abstract])) AND ("Habits"[Mesh] OR "Habits"[Title/Abstract] OR "Habit"[Title/Abstract] OR "long-term"[Title/Abstract] OR "routine"[Title/Abstract]) AND ((humans[Filter]) AND (english[Filter]))

Results: 3,091

Database 2: Embase

Run date: 15/07/2025

(('older adult*':ab,ti,kw OR 'older people':ab,ti,kw OR elderly:ab,ti,kw OR 'older population':ab,ti,kw OR 'senior citizen*':ab,ti,kw) AND ('exercise'/exp OR exercise:ab,ti,kw OR exercises:ab,ti,kw OR 'aerobic exercise':ab,ti,kw OR 'resistance exercise':ab,ti,kw OR 'multi-component exercise':ab,ti,kw OR 'tai chi':ab,ti,kw OR swim:ab,ti,kw OR yoga:ab,ti,kw)) AND ('habit'/exp OR habits:ab,ti,kw OR habit:ab,ti,kw OR 'long term':ab,ti,kw OR routine:ab,ti,kw) AND [humans]/lim AND [english]/lim AND [embase]/lim

Results: 3,333

Database 3: Web of Science

Run date: 15/07/2025

1. AB=("older adult*" OR "older people" OR elderly OR "older population" OR "senior citizen*")
2. AB=(exercise OR exercises OR "aerobic exercise" OR "resistance exercise" OR "multi-component exercise" OR "Tai Chi" OR swim OR yoga)
3. AB=(habit OR habits OR "long-term" OR routine)
4. #1 AND #2 AND #3

Filters: English; Exclude preprints

Results: 3,839

Database 4: EBSCOhost

Run date: 15/07/2025

(AB ("older adult*" OR "older people" OR "elderly" OR "older population" OR "senior citizen*")) AND (AB ("exercise" OR "exercises" OR "aerobic exercise" OR "resistance exercise" OR "multi-component exercise" OR "Tai Chi" OR "swim" OR "Yoga"))
AND (AB ("Habits" OR "Habit" OR "long-term" OR "routine")) AND (LM "Humans")
AND (LA "English")

Results: 100

Database 5: Cochrane Library

Run date: 15/07/2025

"older adult*" OR "older people" OR elderly OR "older population" OR "senior citizen" in Title Abstract Keyword AND exercise OR exercises OR "aerobic exercise" OR "resistance exercise" OR "multi-component exercise" OR "Tai Chi" OR swim OR yoga in Title Abstract Keyword AND habit OR habits OR "long-term" OR routine in Title Abstract Keyword - (Word variations have been searched)

Language: English

Results: 2,176 (2,156 trials and 20 reviews)

Supplementary Table S2A. Characteristics of the included studies

Ref.	Author/year	Country/region	Target population	Aim	Design	Main reported outcomes
[17]	Arkkukangas M et al., 2018	Sweden	114 community-dwelling adults aged >=75 years.	To examine whether behavioral factors, motivational interviewing, readiness to change, fall self-efficacy, and baseline activity habits predicted 1-year adherence to the Otago Exercise Program.	Longitudinal intervention follow-up.	Baseline activity habits predicted exercise and walking adherence. Motivational interviewing was also associated with higher exercise adherence.
[18]	Arkkukangas M et al., 2022	Sweden	200 community-dwelling older adults (mean age approximately 72 years).	To identify factors associated with maintained physical activity after a short multicomponent group exercise intervention during COVID-19 restrictions.	Prospective cohort follow-up.	Baseline activity level, self-rated health, and participation in the higher-challenge group program were associated with activity level at 7-month follow-up.
[19]	Chakrabarti A et al., 2022	Australia	30 adults aged >=60 years with type 1 diabetes using insulin pumps.	To describe physical activity and exercise-related diabetes self-management practices.	Cross-sectional survey.	Participants reported a median of 4 exercise days/week and 60 min/session. Glucose concerns influenced participation and self-management practices.
[20]	Chen Z et al., 2025	China	280 community-dwelling adults aged >=60 years; five survey participants completed semi-structured interviews.	To describe exercise habits and preferences and examine relationships with chronic pain and daily-life impacts.	Mixed-methods: cross-sectional survey and semi-structured interviews.	Participants with chronic pain reported lower exercise participation. Walking was common; social support and a supportive environment were identified as facilitators.
[21]	Fujii Y et al., 2020	Japan	1,520 community-dwelling adults aged >=65 years.	To examine whether exercising with others was associated with incident functional disability and all-cause mortality.	Prospective 5-year cohort study.	Compared with no exercise, exercising with others was associated with lower risks of functional disability and mortality. Adjusted differences versus exercising alone were less consistent.
[22]	Greaney ML et al., 2004	USA	29 adults aged >=60 years.	To explore what older adults considered useful for maintaining healthy eating and exercise habits.	Qualitative focus-group study.	Health maintenance, independence, and fear of illness motivated exercise. Reported strategies included counterconditioning, helping relationships, stimulus control, and self-liberation.
[23]	Hau C et al., 2016	USA	50 Chinese older adults living in the United States (mean age 68.4 years).	To evaluate the Healthy Habits Program on mobility, cognition, and related outcomes.	Quasi-experimental pre-post program evaluation.	Seventy-eight percent achieved >=3 sessions/week. Mobility, global cognition, executive function, and depressive symptoms improved after 6 months.
[24]	Hayashi T et al., 2022	Japan	1,103 community-dwelling adults aged >=65 years.	To examine social frailty and depressive symptoms during the COVID-19 pandemic and the role of home exercise.	Cross-sectional survey.	Social frailty was associated with depressive symptoms. The association was observed among those who did not exercise at home but not among those who did.
[25]	Hernandez-Martinez J et al., 2023	Chile	179 women aged >=60 years.	To examine associations of physical activity habits with cardiometabolic, body-composition, and physical-performance variables.	Analytical cross-sectional study.	Physically inactive participants had less favorable cardiometabolic profiles, body composition, grip strength, timed up-and-go, and walking speed.
[26]	Kawasaki T et al., 2020	Japan	59 community-dwelling adults aged >=65 years with low back pain.	To examine the relationship between grit and self-directed physical training habits.	Cross-sectional study.	Grit was positively correlated with the duration of self-directed exercise and remained a significant factor in multivariable analysis.
[41]	Wang YF et al., 2021	China (Taiwan region)	21 older exercisers and 2 sports instructors.	To explore factors supporting continued exercise among older adults.	Qualitative phenomenological study using semi-structured interviews.	Doctor referral and encouragement initiated participation; tailored gym programs, suitable equipment, a friendly climate, and regular assessment supported continuation. Participants reported better function, self-initiated exercise, and wider social networks.
[27]	Li N et al., 2025	China	7,231 adults aged >=60 years from the Chinese Longitudinal Healthy Longevity Survey.	To examine joint associations of sleep duration and changes in exercise habit with all-cause mortality.	National community-based longitudinal cohort study.	Sustained exercise and transition from inactivity to activity were associated with lower mortality; associations varied by sleep duration and sex.
[28]	Lin WY et al., 2025	China (Taiwan region)	2,545 adults aged >=65 years.	To examine associations of exercise regularity, type, frequency, and duration with frailty.	Cross-sectional study.	Longer daily exercise duration was associated with lower frailty; absence of aerobic exercise was associated with higher pre-frailty.
[29]	Lyu W et al., 2025	Japan	1,288 community-dwelling adults aged >=65 years with 7-	To compare non-exercise physical activity and exercise habits in relation	Prospective cohort study.	Exercise habit and moderate-to-vigorous non-exercise activity were each associated with lower prevalent and incident frailty; their associations did not differ

			year follow-up.	to frailty.		significantly.
[30]	Makino K et al., 2015	Japan	304 community-dwelling older adults (mean age 70.3 years).	To examine associations between individual- versus group-based exercise habits and health-related outcomes.	Cross-sectional questionnaire study with face-to-face administration.	Group-based exercisers had more favorable depressive-symptom, exercise-self-efficacy, and dietary-variety measures than individual exercisers.
[31]	Nakade T et al., 2025	Japan	1,262 hospitalized adults aged >=65 years with heart failure.	To examine associations of pre-admission exercise habits with physical function and post-discharge prognosis.	Longitudinal cohort/post hoc analysis with mortality follow-up.	Patients reporting pre-admission exercise had better physical function and lower post-discharge all-cause mortality after adjustment.
[32]	Nakajima C et al., 2025	Japan	10,152 community-dwelling adults aged >=65 years.	To examine sex differences in associations between exercise habits and loneliness.	Cross-sectional study.	Exercise participation was associated with lower loneliness among men but not women; associations differed by exercise type.
[33]	Otsuka H et al., 2021	Japan	1,596 adults aged 65-84 years.	To examine exercise habits in adolescence and old age in relation to osteoporosis and bone mineral density.	Cross-sectional study.	Women active in both periods had higher bone mineral density and lower osteoporosis prevalence; findings in men were limited.
[34]	Pettersson B et al., 2023	Sweden	18 community-dwelling adults aged >=70 years who maintained exercise during a 12-month digital intervention.	To explore factors enabling maintenance of self-managed digital fall-prevention exercise.	Qualitative grounded-theory study.	A theoretical framework described acting against threats, coordinating strategies to establish routines, and forming habits through cues and evaluation, under the core category of maintaining balance in life.
[35]	Shi H et al., 2024 (MCI)	Japan	1,615 adults aged 65-84 years.	To examine adolescent and old-age exercise habits in relation to mild cognitive impairment, brain volumes, and biomarkers.	Cross-sectional study.	Exercise habits in both adolescence and old age were associated with lower odds of MCI; brain-volume and biochemical-marker differences were not significant.
[36]	Shi H et al., 2024 (depressive symptoms)	Japan	1,629 adults aged 65-84 years.	To examine adolescent and old-age exercise habits in relation to geriatric depressive symptoms.	Cross-sectional study.	Exercise habits in adolescence, old age, or both were associated with lower prevalence of depressive symptoms in men and women.
[37]	Sugisawa H et al., 2020	Japan	739 community-dwelling adults aged >=65 years.	To examine life-course social-network health habits and late-life health habits.	Cross-sectional survey with retrospective life-course recall.	More favorable exercise habits in social networks across the life course were associated with the participant's own late-life exercise habit.
[38]	Tabata H et al., 2023	Japan	1,607 adults aged 65-84 years.	To examine adolescent and old-age exercise habits in relation to sarcopenia and its components.	Cross-sectional study.	Men active in both periods had lower risks of sarcopenia, low muscle mass, and low performance; women active in both had lower risk of low muscle performance.
[39]	Wang S et al., 2021	China	338 nursing-home residents aged >=60 years.	To examine pathways linking exercise with mild cognitive impairment through physical, psychological, and social factors.	Cross-sectional path analysis.	Exercise was associated with MCI through direct and indirect paths involving frailty, depression, sleep quality, and social support; causal direction cannot be established.
[40]	Xu W et al., 2023	China	370 adults aged >=60 years.	To examine the relationship between exercise habits and sleep quality.	Cross-sectional study.	Regular exercise was associated with a higher likelihood of good sleep; age, pain, and self-rated health were also associated with sleep quality.

Note: Reference numbers correspond to the main manuscript. Studies conducted in the Taiwan region were counted under China in the geographical summary.

Supplementary Table S2B. Definitions and measurement approaches used to operationalize exercise habit

Ref.	Author/year	Exercise type	Operational definition	Measurement tool or item	Operationalization category	Validated habit-specific measure
[17]	Arkkukangas M et al., 2018	Otago strength and balance exercises; walking.	Baseline activity habits were analyzed as a predictor; no explicit psychological-habit threshold was reported.	Self-reported baseline activity-habit measure and 52-week exercise/walking adherence records.	Regularity/adherence-based	No
[18]	Arkkukangas M et al., 2022	Multicomponent group exercise emphasizing strength, balance, and challenging functional tasks.	Six activity levels ranged from hardly any activity to strenuous regular exercise several times per week.	Frändin-Grimby Activity Scale, self-administered at follow-up.	Regularity/physical-activity-level proxy	No
[19]	Chakrabarti A et al., 2022	Mainly light-to-moderate activities, including walking and gardening; some aerobic and resistance exercise.	No binary habit definition; exercise days per week, session duration, and activity patterns were described.	Yale Physical Activity Survey plus a study-developed questionnaire on exercise and diabetes self-management.	Regularity/physical-activity-level proxy	No
[20]	Chen Z et al., 2025	Walking, jogging, table tennis, and other self-selected activities.	Exercise habit (yes/no), frequency (<3 vs. >=3 times/week), duration (<30 vs. >=30 min/session), and exercise type were recorded separately; no combined threshold was used.	Author-developed 58-item questionnaire; semi-structured interviews with five participants.	Regularity-based survey with qualitative contextual evidence	No
[21]	Fujii Y et al., 2020	Exercise type not specified; setting was classified as exercising alone or with others.	Exercise habit was defined as exercising at least once per week; exercisers were classified as exercising alone or with others.	Self-administered questionnaire items on exercise frequency and social setting.	Regularity-based	No
[22]	Greaney ML et al., 2004	Exercise type not specified.	No formal frequency, duration, or automaticity criterion was applied.	Qualitative focus-group discussions about adopting and maintaining exercise.	Qualitative process evidence	No
[23]	Hau C et al., 2016	Cardiovascular, strength, flexibility, and group fitness activities.	The program exercise goal was at least three sessions per week; adherence was assessed during the program.	Program participation/adherence records and pre-post outcome assessments.	Regularity/adherence-based	No
[24]	Hayashi T et al., 2022	Home exercise; specific activity types were not reported.	Home exercise was represented by weekly exercise frequency.	Single self-reported item on home-exercise frequency in the previous week.	Regularity-based single-item measure	No
[25]	Hernandez-Martinez J et al., 2023	Moderate- and vigorous-intensity physical activity.	Participants were classified as physically active if they met weekly moderate- or vigorous-intensity activity recommendations.	Self-reported weekly physical-activity dose used to classify physically active versus inactive participants.	Physical-activity-dose proxy labeled as habit	No
[26]	Kawasaki T et al., 2020	Aerobic exercise and muscle-strengthening exercise.	Self-directed exercise was defined as moderate-or-higher intensity exercise more than twice weekly for more than 30 min/session; maintenance duration was recorded.	Self-reported exercise frequency, intensity, session duration, type, and duration of maintaining the pattern.	Regularity/duration-based	No
[41]	Wang YF et al., 2021	Gym-based, individually tailored exercise programs.	No formal frequency, duration, or automaticity threshold was applied; participants had experience with continued exercise.	Semi-structured interviews analyzed using a qualitative phenomenological approach.	Qualitative process evidence	No
[27]	Li N et al., 2025	Exercise type not specified.	Regular exercise status was self-reported across waves and categorized as inactive, active, inactive-to-active, or active-to-inactive.	Repeated self-report item on regular exercise in the Chinese Longitudinal Healthy Longevity Survey.	Regularity/change-based	No
[28]	Lin WY et al., 2025	Aerobic and other self-reported exercise types.	Exercise was characterized by regularity, type, days/week, and daily duration.	Structured self-report questionnaire with categorical frequency and duration response options.	Regularity/dose-based	No
[29]	Lyu W et al., 2025	Exercise type not specified; non-exercise activities included work, household, and other daily activities.	Exercise habit was defined as at least 30 min/day, more than twice weekly, and continued for more than one year.	Self-reported exercise-engagement item at baseline/follow-up examinations; non-exercise activity measured with the Global Physical Activity Questionnaire.	Regularity/continuity-based	No
[30]	Makino K et al., 2015	Specific exercise types were not reported; setting was individual or group based.	Exercise habit was defined as exercise more than three times weekly for at least 30 min/session; setting was individual or group based.	Face-to-face interview and self-reported questionnaire.	Regularity-based	No
[31]	Nakade T et al., 2025	Walking, moderate-intensity activity, and vigorous-intensity activity.	Pre-admission exercise was assessed using usual-week bouts: walking/moderate activity lasting at least 30 min and vigorous activity lasting at least 20 min.	Self-reported pre-admission exercise questionnaire covering walking, moderate activity, and vigorous activity.	Regularity/dose-based	No
[32]	Nakajima C et al., 2025	Fourteen types, including walking, jogging, swimming, cycling, racket sports, golf, dance, strength training, yoga, tai chi, hiking, gymnastics, group exercise, and team games.	Participants reported exercise participation during the previous year across 14 activity types; no minimum frequency, duration, or automaticity threshold was specified.	Questionnaire asking whether each of 14 exercise types had been performed during the previous year.	Participation/type-based proxy	No

[33]	Otsuka H et al., 2021	Adolescent sports-club activity; current exercise type not specified.	Adolescent habit was school sports-club participation; old-age habit was a yes/no report of current exercise habits.	Self-administered questionnaire items on adolescent sports-club participation and current exercise habits.	Participation/current-regularity proxy	No
[34]	Pettersson B et al., 2023	Safe Step app-based balance and strength exercises.	Eligibility required at least 60 min/week of exercise during the final three intervention months; habit development was explored qualitatively.	Semi-structured interviews analyzed using grounded theory; no formal habit scale.	Qualitative process evidence	No
[35]	Shi H et al., 2024 (MCI)	Adolescent school sports-club activity; current exercise type not specified.	Adolescent habit was school sports-club participation; old-age habit was a yes/no report of current exercise habits.	Self-administered questionnaire items on adolescent sports-club participation and current exercise habits.	Participation/current-regularity proxy	No
[36]	Shi H et al., 2024 (depressive symptoms)	Adolescent school sports-club activity; current exercise type not specified.	Adolescent habit was school sports-club participation; current habit was exercise more than twice weekly for at least 30 min/session.	Self-administered questionnaire items on adolescent sports-club participation and current exercise frequency/duration.	Participation/regularity-based	No
[37]	Sugisawa H et al., 2020	Exercise type not specified.	Exercisers were defined as exercising more than 30 min twice weekly or more than 20 min three times weekly for more than one year.	Self-administered questionnaire with retrospective life-course social-network and personal health-habit items.	Regularity/continuity-based	No
[38]	Tabata H et al., 2023	Aerobic/cardiovascular and strength/resistance activities.	Adolescent habit was school sports-club participation; current habit was exercise more than twice weekly for at least 30 min/session.	Self-administered questionnaire items on adolescent sports-club participation and current exercise frequency/duration.	Participation/regularity-based	No
[39]	Wang S et al., 2021	Exercise type not specified.	Regular exercise was defined as at least 30 min/day on at least three days/week.	Single self-reported regular-exercise item within the study questionnaire.	Regularity-based	No
[40]	Xu W et al., 2023	Exercise type not specified.	Exercise habit was defined as at least 30 min/session on at least three days/week.	Single dichotomous self-reported exercise-habit item.	Regularity-based	No

Note: Operationalization categories were assigned in this review. No included study used a validated habit-specific automaticity instrument such as the Self-Report Habit Index or Self-Report Behavioral Automaticity Index. Regularity-based measures capture repeated or sustained exercise but do not directly establish cue-dependent automaticity.

Supplementary Table S3. Methodological quality appraisal using the Mixed Methods Appraisal Tool (MMAT), version 2018

Ratings: Y, Yes; N, No; CT, Can't tell. All studies met the two MMAT screening questions (clear research question and data suitable to address the question). In accordance with MMAT guidance, no overall numerical score was calculated. This first-pass appraisal should be independently verified by a second author before submission.

Supplementary Table S3A. Qualitative studies

Ref.	Author/year	1.1	1.2	1.3	1.4	1.5	Key methodological considerations
[22]	Greaney ML et al., 2004	Y	Y	Y	Y	CT	Focus-group methods and findings were relevant, but full coherence across sampling, analysis, and interpretation was not completely reported.
[41]	Wang YF et al., 2021	Y	Y	Y	Y	CT	Phenomenological interviews provided relevant process evidence; researcher positioning and the audit trail were not fully clear.
[34]	Pettersson B et al., 2023	Y	Y	Y	Y	Y	Grounded-theory design, iterative analysis, and development of the conceptual categories were clearly aligned.

Criteria: 1.1 appropriate qualitative approach; 1.2 adequate qualitative data collection; 1.3 findings adequately derived from data; 1.4 interpretation sufficiently substantiated by data; 1.5 coherence among data sources, collection, analysis, and interpretation.

Supplementary Table S3B. Quantitative non-randomized studies

Ref.	Author/year	3.1	3.2	3.3	3.4	3.5	Key methodological considerations
[17]	Arkkukangas M et al., 2018	CT	CT	CT	Y	Y	Selected intervention participants; baseline activity-habit measurement and attrition details require cautious interpretation.
[18]	Arkkukangas M et al., 2022	N	Y	CT	Y	Y	Intervention participants were not population-representative; follow-up attrition was not fully resolved.
[21]	Fujii Y et al., 2020	CT	CT	Y	Y	Y	Low valid response rate and unvalidated self-reported exercise classification; residual confounding remained possible.
[23]	Hau C et al., 2016	N	Y	CT	N	Y	Small single-group pre-post evaluation without a comparator; selection and confounding limit attribution of change.
[24]	Hayashi T et al., 2022	CT	CT	CT	Y	Y	Cross-sectional self-report data; temporality and non-response bias were uncertain.
[25]	Hernandez-Martinez J et al., 2023	N	CT	Y	Y	Y	Recruitment from community activity settings and self-reported activity limited representativeness and exposure validity.
[26]	Kawasaki T et al., 2020	N	CT	Y	CT	Y	Small selected sample with low back pain; self-reported exercise duration and limited confounder control.
[27]	Li N et al., 2025	Y	CT	CT	Y	Y	National cohort and adjusted survival analysis, but regular exercise was self-reported, and longitudinal missingness may affect estimates.
[28]	Lin WY et al., 2025	CT	CT	CT	Y	Y	Cross-sectional exercise-dose categories were self-reported; sampling and missing-data handling were incompletely reported.
[29]	Lyu W et al., 2025	CT	Y	CT	Y	Y	Prospective design and validated frailty assessment; selection through follow-up and self-reported exercise remain concerns.
[30]	Makino K et al., 2015	CT	CT	CT	CT	Y	Cross-sectional self-report study; representativeness, missingness, and residual confounding were uncertain.
[31]	Nakade T et al., 2025	CT	Y	Y	Y	Y	Hospital-based heart-failure cohort limits generalizability, although outcomes and adjusted longitudinal analyses were appropriate.
[32]	Nakajima C et al., 2025	CT	CT	CT	Y	Y	Large cross-sectional sample, but exercise was broadly self-reported, and non-response/missingness were not fully clear.
[33]	Otsuka H et al., 2021	CT	CT	Y	Y	Y	Volunteer cohort and retrospective adolescent exercise recall; objective bone outcomes and adjusted analyses strengthened the study.
[35]	Shi H et al., 2024 (MCI)	CT	CT	Y	Y	Y	Cross-sectional volunteer sample and retrospective exercise recall prevented temporal inference.
[36]	Shi H et al., 2024 (depressive symptoms)	CT	CT	Y	Y	Y	Cross-sectional volunteer sample and retrospective exercise recall; causality and temporal order cannot be established.
[37]	Sugisawa H et al., 2020	Y	CT	CT	Y	Y	Population survey with adjusted analyses, but retrospective life-course recall and non-response may introduce bias.
[38]	Tabata H et al., 2023	CT	CT	Y	Y	Y	Volunteer sample and retrospective adolescent exercise recall; objective sarcopenia outcomes strengthened measurement.
[39]	Wang S et al., 2021	N	CT	Y	CT	Y	Convenience sample from nursing homes and cross-sectional path analysis do not establish mediation or causal direction.
[40]	Xu W et al., 2023	CT	CT	Y	Y	Y	Single-item exercise-habit exposure and cross-sectional design; sleep outcome was assessed with a validated instrument.

Criteria: 3.1 participants representative of the target population; 3.2 appropriate measurements of exposure/intervention and outcomes; 3.3 complete outcome data; 3.4 confounders accounted for in design and analysis; 3.5 exposure/intervention occurred or was administered as intended.

Supplementary Table S3C. Quantitative descriptive study

Ref.	Author/year	4.1	4.2	4.3	4.4	4.5	Key methodological considerations
[19]	Chakrabarti A et al., 2022	N	N	CT	CT	Y	Small clinical sample of insulin-pump users; study-developed self-report items supplemented by a validated activity survey.

Criteria: 4.1 relevant sampling strategy; 4.2 sample representative of the target population; 4.3 appropriate measurements; 4.4 low risk of non-response bias; 4.5 appropriate statistical analysis.

Supplementary Table S3D. Mixed-methods study: Chen Z et al., 2025 [20]

Component	Criterion 1	Criterion 2	Criterion 3	Criterion 4	Criterion 5	Key methodological considerations
Quantitative descriptive component	N	N	CT	CT	Y	Snowball sampling, limited diversity, and author-developed exercise items; some pain and psychological outcome tools were validated.
Qualitative component	CT	CT	CT	CT	CT	Only five selected interviewees; qualitative sampling, analytic procedure, saturation, and evidentiary linkage were insufficiently detailed.
Mixed-methods integration	CT	N	CT	CT	N	No clear mixed-methods rationale or explicit integration procedure; quantitative and qualitative findings were largely reported in parallel.

Quantitative component criteria 4.1-4.5 and qualitative component criteria 1.1-1.5 are defined above. Mixed-methods criteria: 5.1 adequate rationale for using mixed methods; 5.2 effective integration of components; 5.3 integrated outputs adequately interpreted; 5.4 divergences and inconsistencies adequately addressed; 5.5 each component adheres to the quality criteria of its methodological tradition.

Summary of the critical appraisal

The appraisal identified recurring limitations in participant representativeness, reliance on self-reported and non-standardized exercise-habit measures, incomplete reporting of non-response or attrition, and residual confounding. Cross-sectional studies could identify associations but could not establish temporal direction. The qualitative studies contributed relevant contextual evidence, although reporting of analytic coherence was not always complete. The mixed-methods study had limited explicit integration between its quantitative and qualitative components. These findings support interpreting the proposed conceptual map as an organizational and hypothesis-generating synthesis rather than a validated sequential or causal pathway.

Supplementary Table S4. Quantitative findings stratified by study design and exercise-habit operationalization

The table reports the most fully adjusted estimate where available; otherwise, the principal quantitative result is shown. Null, adverse, subgroup-specific, and attenuated findings are presented alongside favorable findings. Studies are grouped first by design and outcome type, and the exercise-habit operationalization used in each study is shown explicitly. Estimates were not statistically pooled because exposure definitions, comparisons, and outcomes were heterogeneous. No quantitative study used a validated habit-specific automaticity measure.

Supplementary Table S4A. Longitudinal, follow-up, and quasi-experimental quantitative findings

Ref.	Author/year	Operationalization category	Exposure/comparison	Outcome	Most fully adjusted or main result	Finding pattern	Methodological interpretation
**Longitudinal health-related outcomes**							
[21]	Fujii Y et al., 2020	Regularity/social-setting-based	Exercising with others vs. no exercise; with others vs. alone	Incident functional disability and all-cause mortality over 5 years	With others vs. no exercise: disability HR 0.59 (95% CI 0.40-0.88) and mortality HR 0.40 (0.24-0.66). With others vs. alone after adjustment: disability HR 0.74 (0.48-1.14) and mortality HR 0.67 (0.38-1.17).	Mixed	Prospective cohort. Comparisons with no exercise were favorable, but adjusted comparisons with exercising alone were null.
[27]	Li N et al., 2025	Regularity/change-based	Active, inactive-to-active, and active-to-inactive vs. inactive	All-cause mortality	Fully adjusted: active HR 0.74 (95% CI 0.66-0.83), inactive-to-active HR 0.88 (0.80-0.97), and active-to-inactive HR 1.10 (0.99-1.22). Among men with short sleep, active HR 1.585 (1.019-2.465); among women with short sleep, active HR 0.816 (0.527-1.263).	Mixed/adverse subgroup	National longitudinal cohort. Overall estimates were favorable, but sex- and sleep-specific findings were heterogeneous.
[29]	Lyu W et al., 2025	Regularity/continuity-based	Exercise habit (EH), non-exercise physical activity (NEPA), both, vs. neither	Prevalent and incident frailty	Prevalent frailty: EH only OR 0.21 (95% CI 0.11-0.41), NEPA only OR 0.29 (0.16-0.52), and both OR 0.21 (0.12-0.36). Incident frailty: EH only HR 0.51 (0.29-0.90), NEPA only HR 0.55 (0.33-0.92), and both HR 0.42 (0.25-0.70).	Favorable/mixed	Prospective cohort. EH and NEPA were each associated with frailty, but their estimates did not differ significantly and were not clearly additive.
[31]	Nakade T et al., 2025	Regularity/dose-based	Pre-admission exercise habit vs. no exercise habit	Post-discharge all-cause mortality and physical function	Adjusted mortality HR 0.75 (95% CI 0.58-0.98; p=0.035). At discharge, the exercise-habit group had better grip strength, gait speed, chair-stand performance, SPPB, and 6-min walk distance than the no-exercise group (all p<0.001).	Favorable	Hospital-based longitudinal cohort. Mortality was adjusted; physical-function comparisons were between groups and were not presented as adjusted effect estimates.
**Longitudinal exercise-maintenance and adherence outcomes**							
[17]	Arkkukangas M et al., 2018	Regularity/adherence-based	Baseline activity habits and motivational-interviewing allocation	52-week exercise and walking adherence	Baseline activity habits predicted exercise adherence, OR 3.39 (95% CI 1.38-8.32), and walking adherence, OR 6.11 (2.34-15.94). Motivational interviewing predicted exercise adherence, OR 2.47 (1.11-5.49).	Favorable	Longitudinal intervention follow-up. Outcomes concerned adherence rather than health outcomes or automaticity.
[18]	Arkkukangas M et al., 2022	Physical-activity-level/maintenance proxy	Baseline activity level, self-rated health, and higher-challenge group program	Activity level at 7-month follow-up	Baseline activity level OR 2.83 (95% CI 1.20-6.71), self-rated health OR 2.80 (1.42-5.53), and higher-challenge group OR 2.04 (1.04-4.00).	Favorable	Prospective follow-up of intervention participants. Estimates concerned maintenance of activity rather than habit automaticity.
**Quasi-experimental program outcome**							
[23]	Hau C et al., 2016	Program participation/adherence-based	Participation in the Healthy Habits Program; goal >=3 sessions/week	Adherence, mobility, cognition, executive function, and depressive symptoms at 6 months	Seventy-eight percent achieved >=3 sessions/week. SPPB increased from 10.3 to 11.1 (p=0.01), and MMSE from 26.0 to 27.8 (p=0.001); other reported outcomes also improved (p<0.05).	Favorable	Single-group pre-post evaluation without a comparator; observed changes cannot be attributed causally to the program.

Supplementary Table S4B. Cross-sectional and mixed-methods quantitative findings

Ref.	Author/year	Operationalization category	Exposure/comparison	Outcome	Most fully adjusted or main result	Finding pattern	Methodological interpretation
**Physical, frailty, cardiometabolic, and musculoskeletal outcomes**							
[25]	Hernandez-Martinez J et al., 2023	Physical-activity-dose proxy labeled as habit	Physically inactive vs. physically active	Cardiometabolic, body-composition, and physical-performance outcomes	Physically inactive participants had higher odds of hyperglycemia, OR 4.70 (95% CI 2.16-10.90), low HDL, OR 2.13 (1.04-4.37), excess body fat, OR 4.33 (2.00-9.37), low fat-free mass, OR 2.22 (1.08-4.58), poor TUG, OR 5.60 (2.44-12.60), and slow gait, OR 5.52 (2.46-12.30).	Favorable association with activity	Analytical cross-sectional study of older women. Logistic regressions were not described as multivariable-adjusted.
[28]	Lin WY et al., 2025	Regularity/dose-based	Exercise duration and aerobic-exercise participation	Frailty and pre-frailty	Compared with <15 min/day, 31-60 min/day and >60 min/day were reported as corresponding to 59% and 69% lower frailty, respectively. Absence of aerobic exercise was associated with 34% higher pre-frailty.	Favorable/dose-related	Cross-sectional self-report study. Results were reported as relative percentage differences; temporal direction and residual confounding cannot be resolved.
[33]	Otsuka H et al., 2021	Participation/current-regularity proxy	Active in adolescence and old age (AA) vs. inactive in both periods (NN)	Osteoporosis prevalence and bone mineral density	Women: lumbar osteoporosis OR 0.65 (95% CI 0.42-1.00; p=0.049) and hip osteoporosis OR 0.70 (0.46-1.06; p=0.089). Osteoporosis associations in men were not significant.	Mixed/sex-specific	Cross-sectional study with retrospective adolescent exposure. Objective bone outcomes strengthened measurement, but recall and selection bias remained possible.
[38]	Tabata H et al., 2023	Participation/regularity-based	Exercise in adolescence and old age (AA) vs. inactive in both periods (NN)	Sarcopenia, low muscle mass, and low muscle performance	Men in the AA group: sarcopenia OR 0.29 (95% CI 0.09-0.95), low muscle mass OR 0.21 (0.09-0.52), and low muscle performance OR 0.52 (0.28-0.97). Women: low muscle performance OR 0.48 (0.27-0.84); sarcopenia and low muscle mass were not significant.	Mixed/sex-specific	Cross-sectional study with retrospective adolescent exposure. Associations differed by sex and outcome.
**Psychological, cognitive, social, and sleep outcomes**							
[24]	Hayashi T et al., 2022	Regularity-based single-item measure	Social frailty vs. no social frailty, stratified by home exercise	Depressive symptoms	Overall OR 1.80 (95% CI 1.16-2.79). No home exercise: OR 2.10 (1.14-3.84). Home exercise: OR 1.50 (0.79-2.85), not statistically significant.	Mixed/stratified	Cross-sectional study. Home exercise was measured with a single self-reported item and cannot be interpreted as a causal moderator.
[30]	Makino K et al., 2015	Regularity/social-setting-based	Group-based vs. individual-based exercise habit	Depressive symptoms, exercise self-efficacy, and dietary variety	Adjusted for age and sex: GDS OR 0.76 (95% CI 0.65-0.90), exercise self-efficacy OR 1.26 (1.11-1.44), and food-frequency score OR 0.93 (0.85-1.02), not statistically significant.	Mixed	Cross-sectional study. Two outcomes were favorable, whereas dietary variety was null after adjustment.
[32]	Nakajima C et al., 2025	Participation/type-based proxy	Exercise participation vs. no participation, stratified by sex	Loneliness	Men OR 0.75 (95% CI 0.65-0.88); women OR 0.96 (0.82-1.13), not statistically significant. Associations also differed by exercise type.	Mixed/sex-specific	Large cross-sectional study. The exposure captured participation across exercise types rather than frequency, duration, or automaticity.
[35]	Shi H et al., 2024 (MCI)	Participation/current-regularity proxy	Exercise in adolescence and/or old age vs. inactive in both periods	MCI, regional brain volumes, and biomarkers	AA vs. NN: MCI OR 0.62 (95% CI 0.41-0.94). Old-age only vs. NN OR 0.72 (0.48-1.08), and adolescence only vs. NN OR 0.77 (0.49-1.19), both not significant. Brain-volume and biomarker differences were not significant.	Mixed	Cross-sectional study. The favorable MCI association was confined to the both-period group, while proposed mechanistic outcomes were null.
[36]	Shi H et al., 2024 (depressive symptoms)	Participation/regularity-based	Exercise in adolescence and/or old age vs. inactive in both periods	Geriatric depressive symptoms	Fully adjusted ORs: men, old-age only 0.48 (95% CI 0.26-0.90), adolescence only 0.51 (0.31-0.86), and both periods 0.45 (0.25-0.81); women, 0.63 (0.40-0.97), 0.59 (0.40-0.89), and 0.63 (0.40-0.99), respectively.	Favorable but non-additive	Cross-sectional study with retrospective recall. Activity in both periods did not show a clear additive or synergistic association.
[39]	Wang S et al., 2021	Regularity-based	Regular exercise	MCI and paths through frailty, depression, sleep, and social support	Direct and indirect paths between exercise and MCI through frailty, depression, sleep quality, and social support were reported as statistically significant (p<0.05).	Favorable path associations	Cross-sectional path analysis cannot establish mediation, temporal order, or causal mechanisms.
[40]	Xu W et al., 2023	Regularity-based	Exercise habit vs. no exercise habit	Good sleep quality	Adjusted OR 2.09 (95% CI 1.12-3.88; p<0.05); average marginal effect +12.66%. Propensity-score analyses were directionally consistent.	Favorable	Cross-sectional single-item exposure. Propensity-score adjustment does not eliminate unmeasured confounding or establish temporality.
**Exercise participation, maintenance, and contextual outcomes**							
[19]	Chakrabarti A et al., 2022	Physical-activity-level proxy	Exercise days, duration, and diabetes self-management practices	Exercise pattern and glucose-management concerns	Median 4 exercise days/week (IQR 3-6) and 60 min/session (IQR 40-90); 70% reported glucose-related concerns influencing participation. No adjusted exercise-health association was examined.	Descriptive	Small cross-sectional clinical sample. The study provided descriptive evidence on participation and self-management.
[20]	Chen Z et al., 2025	Regularity-based survey with qualitative context	Chronic pain vs. no chronic pain; exercise frequency and duration	Exercise participation and preferences	Exercise-habit yes/no: p=0.629. Exercising >=3 times/week: 48.66% with pain vs. 64.52% without pain (p=0.012). Session duration >=30 min: p=0.423.	Mixed	Mixed-methods survey. Quantitative estimates were unadjusted group comparisons and did not establish directionality.
[26]	Kawasaki T et al., 2020	Regularity/duration-based	Grit score	Duration of self-directed exercise habit	Grit correlated with duration of self-directed exercise, r=0.49 (p<0.001), and remained a significant correlate in multiple regression.	Favorable	Small selected cross-sectional sample with low back pain. The outcome was exercise maintenance duration rather than a health outcome.
[37]	Sugisawa H et al., 2020	Regularity/continuity-based	Life-course exercise habits in social networks	Participant's late-life exercise habit	Indirect pathway coefficient 0.125 (p<0.001); shift toward poorer network exercise habits beta=-0.235 (p<0.05); lifetime absence of better network habits beta=-0.395 (p<0.01).	Mixed directional associations	Cross-sectional survey with retrospective life-course recall. The outcome was exercise habit rather than health.

Abbreviations: AA, active in adolescence and old age; CI, confidence interval; EH, exercise habit; GDS, Geriatric Depression Scale; HDL, high-density lipoprotein; HR, hazard ratio; IQR, interquartile range; MCI, mild cognitive impairment; MMSE, Mini-Mental State Examination; NEPA, non-exercise physical activity; NN, inactive in adolescence and old age; OR, odds ratio; SPPB, Short Physical Performance Battery; TUG, timed up-and-go. Operationalization categories were assigned in this review using the definitions and measures reported in each article.

Checklist S1. PRISMA-ScR checklist

Section/topic	Item	Checklist item	Manuscript location
TITLE	1	Title. Identify the report as a scoping review.	Main manuscript, title.
ABSTRACT	2	Structured summary. Provide a structured summary that includes background, objectives, eligibility criteria, sources of evidence, charting methods, results, and conclusions related to the review questions and objectives.	Main manuscript, Abstract.
INTRODUCTION	3	Rationale. Describe the rationale for the review in the context of what is already known. Explain why the review questions/objectives lend themselves to a scoping review approach.	Main manuscript, Introduction.
INTRODUCTION	4	Objectives. Provide an explicit statement of the questions and objectives being addressed with reference to their key elements.	Main manuscript, Introduction (review questions).
METHODS	5	Protocol and registration. Indicate whether a review protocol exists; state if and where it can be accessed, and provide registration information if available.	Main manuscript, Materials and Methods, Search strategy.
METHODS	6	Eligibility criteria. Specify characteristics of the sources of evidence used as eligibility criteria and provide a rationale.	Main manuscript, Materials and Methods, Inclusion and exclusion criteria.
METHODS	7	Information sources. Describe all information sources in the search and the date of the most recent search.	Main manuscript, Materials and Methods, Search strategy; Supplementary Table S1.
METHODS	8	Search. Present the full electronic search strategy for at least one database, including any limits used, so that it could be repeated.	Main manuscript, Materials and Methods, Search strategy; Supplementary Table S1.
METHODS	9	Selection of sources of evidence. State the process for selecting sources of evidence included in the scoping review.	Main manuscript, Materials and Methods, Study selection.
METHODS	10	Data charting process. Describe the methods of charting data from included sources and any processes for obtaining and confirming data.	Main manuscript, Materials and Methods, Data extraction.
METHODS	11	Data items. List and define all variables for which data were sought and any assumptions or simplifications made.	Main manuscript, Materials and Methods, Data extraction; Supplementary Tables S2A, S2B, S4A, and S4B.
METHODS	12	Critical appraisal of individual sources of evidence. If done, provide a rationale for conducting a critical appraisal and describe the methods used.	Main manuscript, Materials and Methods, Critical appraisal; Supplementary Tables S3A-S3D.
METHODS	13	Synthesis of results. Describe the methods of handling and summarizing the data that were charted.	Main manuscript, Materials and Methods, Data synthesis; Supplementary Tables S4A and S4B.
RESULTS	14	Selection of sources of evidence. Give numbers of sources screened, assessed for eligibility, and included, with reasons for exclusions at each stage, ideally using a flow diagram.	Main manuscript, Results, Study selection and characteristics; Figure 1.
RESULTS	15	Characteristics of sources of evidence. For each source of evidence, present characteristics for which data were charted and provide citations.	Main manuscript, Results, Study selection and characteristics; Figure 2; Supplementary Tables S2A and S2B.
RESULTS	16	Critical appraisal within sources of evidence. If done, present data on critical appraisal of included sources of evidence.	Main manuscript, Results, Methodological quality of the included studies; Supplementary Tables S3A-S3D.
RESULTS	17	Results of individual sources of evidence. For each included source of evidence, present the relevant data that were charted.	Supplementary Tables S2A, S2B, S4A, and S4B.
RESULTS	18	Synthesis of results. Summarize and/or present the charting results as they relate to the review questions and objectives.	Main manuscript, Results; Supplementary Tables S4A and S4B.
DISCUSSION	19	Summary of evidence. Summarize the main results, including an overview of concepts, themes, and types of evidence available; link to the review questions and objectives.	Main manuscript, Discussion.
DISCUSSION	20	Limitations. Discuss the limitations of the scoping review process.	Main manuscript, Limitations.
DISCUSSION	21	Conclusions. Provide a general interpretation of the results with respect to the review questions and objectives, as well as potential implications and/or next steps.	Main manuscript, Conclusions.
FUNDING	22	Funding. Describe sources of funding for the included sources of evidence, as well as sources of funding for the scoping review; describe the role of funders.	Main manuscript, Funding statement.

Abbreviations: PRISMA-ScR, Preferred Reporting Items for Systematic Reviews and Meta-Analyses Extension for Scoping Reviews; OSF, Open Science Framework.
